# Combined method of identification of free oscillations of perforated riffled plates

**DOI:** 10.1038/s41598-025-06614-5

**Published:** 2025-07-04

**Authors:** Serhii Kharchenko, Sylwester Samborski, Lukasz Kloda, Farida Kharchenko

**Affiliations:** 1 Faculty of Mechanical Engineering, Lublin University of Technology, Lublin, Poland; 2 Department of Agricultural Engineering, Sumy National Agrarian University, Sumy, Ukraine

**Keywords:** Computational mechanics, Design, Experimental mechanics, Structures, Vibration, Mechanical engineering, Civil engineering

## Abstract

Presented experimental–numerical method of prediction of modal characteristics of rectangular perforated plates with riffles. The technique allows to analyze the oscillation frequency of plates with holes and stamped riffles. The methodology is based on numerical modeling by FE-methods and experiments to obtain the regularities of changes in the natural oscillation frequency of plate. The methodology involves numerical modelling in Abaqus, the adequacy of which is experimentally verified on a vibrometer PSV-500. Experimental studies are conducted on basic plates with elongated holes measuring 3.2 × 20 mm and 5 × 20 mm, as well as riffled plates with similar holes and different riffles. As a result of the research, dependences of the natural oscillation frequency of riffled perforated plates on their parameters are obtained in comparison with unriffled surfaces. The analysis of the obtained models is carried out and the levels of influence of significant parameters of riffled plates on their natural frequency are determined. The dependences of the natural oscillation frequency of plates on the plate thickness, height, pitch and riffles arrangement, as well as the equations describing them are obtained. The greatest influence on the natural frequencies of riffled perforated plates is exerted by: the plate thickness, the pitch arrangement and the riffle’s height. The obtained regularities of the natural oscillation frequencies of riffled perforated plates may allow for predicting the appearance of deformations in the form of cracks between the holes and determining their durability

**Supplementary Information:**

The online version contains supplementary material available at 10.1038/s41598-025-06614-5.

## Introduction

The perforated vibration surfaces (PVS) are designed to separate loose medium components by size across various industries, including agricultural^1^, food^2^, pharmaceutical and chemical^3^, and mining^4,5^.

Vibration intensifies segregation of loose medium components and their sifting into the holes, but negatively affects the reliability of PVS design^6,7^. The second negative reliability factor is change of PVS thickness due to the abrasive action of loose material particles during its movement. Reduction of PVS thickness and the presence of periodic oscillations lead at a certain point to a decrease of the structure stiffness and the appearance of damage between the holes in the form of cracks^8^. It should be noted that the presence of even partial damage in the form of cracks between PVS holes leads to loss of quality of the technological process of separation due to mixing of descent and passage particles. Furthermore, after the appearance of a crack, there is an intensity in the further deformation of the structure.

The technological indicators of separating equipment operation are determined by the working area of PVS sifting. The forced vibration of this surface can coincide with its natural oscillation frequency, which leads to resonance phenomena and deformations of the structure^9^. A negative component is the variability of PVS thickness and the distinctiveness of vibration parameters for different separating machines.

The application of new PVS designs with intensifiers, activators, and riffle-ribs has demonstrated a significant increase in the efficiency and quality of particle sifting through the holes, compared to typical (serial) surfaces with round, rectangular and triangular holes^10–12^. The riffles, arranged in rows or in checkerboard pattern between the perforations, are created through the cold stamping of sheet metal. This design with vibration has shown positive results in the separation of loose mediums due to the additional orientation of the flat particles into the holes. The riffle lifts the flat component and aligns it through the thickness into the holes However, a comprehensive study of the application of such designs is limited due to the lack of data on their reliability. Preliminary studies of PVS with holes of complex geometry—in the form of three-petal epicycloid instead of triangular-shaped holes – have shown a significant increase in reliability due to reduced stress concentration between the holes^13^.

In the context of the set task, PVS will be considered as homogeneous rectangular plates of constant thickness and material properties from which they are made. The research on reliability for such plates consists of the following stages^13^: determination of thickness wear patterns; identification of the natural oscillations; determination of stress concentrators and obtaining durability patterns. At this stage, we will carry out the identification of the natural oscillation frequency depending on the parameters of the vibration plate.

Determination of the natural oscillation frequencies of parts is possible by analytical^14,15^, experimental^16,17^ and numerical^18–20^ methods. Application of analytical methods is not difficult for parts with academic geometry: flat plates^21^, beams^22,23^, shells^24^, etc. Geometric complication of parts: the presence of curved sections, surfaces, holes, ribs and other structural elements leads to more complicated calculations, increased labor intensity and reduced accuracy. This requires the search for new combined research methods.

One of the most common and effective methods for analyzing the reliability of structures, especially those subjected to vibrations during operation, is modal^25,26^. They are based on the analysis of the natural frequencies and vibration mode shapes of structures, they are available for testing in laboratory (production) conditions. Among the most popular methods based on modal analysis are the modal displacement and modal curvature methods^27–29^ and modal strain energy methods^30^.

Among popular experimental methods for identification of the natural oscillation frequencies of PVS is the use of the Simcenter Testlab measuring complex 2019.1^23,25^. The disadvantages of most experimental methods include insufficient sensitivity to dynamics and structural damage, the requirement for a considerable number of sensors, and the necessity to consider a set of boundary and initial conditions^31,32^. This is a significant limitation for their use on many designs, including PVS, leads to time consuming experimental tests as well as reduced measurement accuracy.

The development of experimental hardware and software provides new opportunities for measuring modal displacements with high accuracy. For example, the use of non-contact methods of modal analysis, for example, scanning with a laser Doppler vibrometer, showed a positive result in terms of wide functionality, the possibility of use on various structures, and sufficient accuracy^33–37^. The high efficiency of this method is ensured by minimizing the influence of external factors (including human factors), the use of a significant number of scanning points and repetitions.

**Fig. 1:**
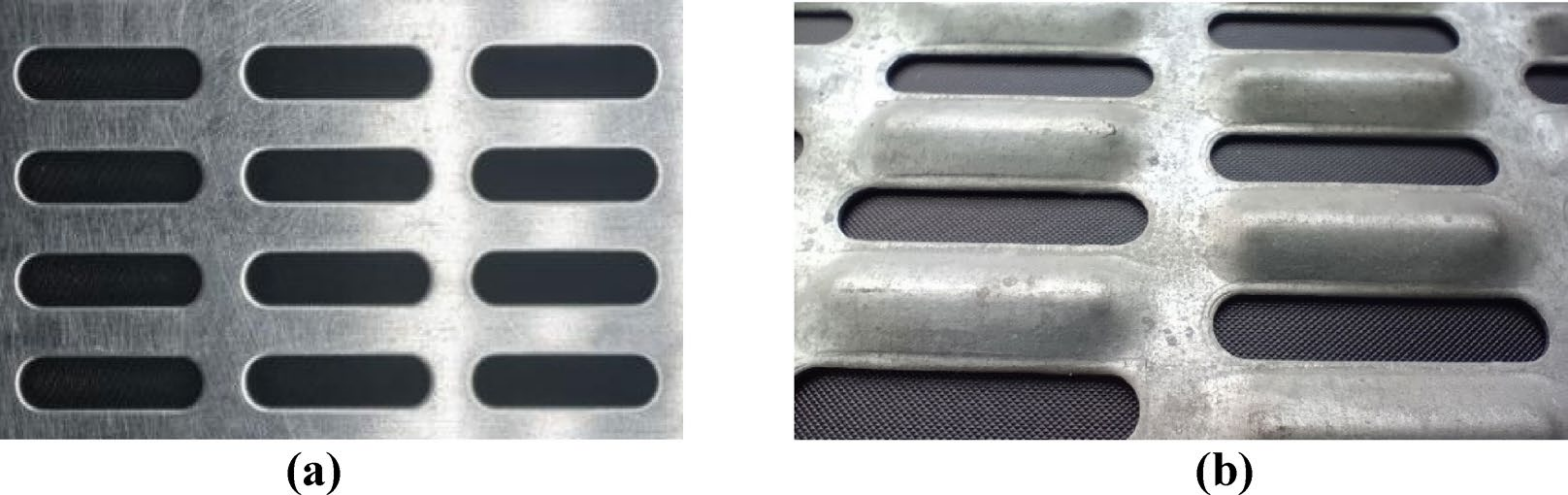
Experimental samples of perforated vibration surfaces. (**a**) basic; (**b**) riffled.

On the one hand, the presence of many holes and riffles on the plate (Fig. 1) complicates the analytical analysis, and on the other hand, through the application of numerical methods, allows to obtain the patterns of changes in the natural oscillations of PVS from its parameters with minimal expenditure of resources and time.

Among the numerical methods for studying perforated plates, it is worth mentioning the finite element method (FEM), which is widely used for scientific research and solving practical engineering problems^25,38,39^. The accuracy of FEM depends on: boundary and initial conditions, values and coordinates of applied loads, technological deviations of the production of perforated surfaces, which require similarity to real processes.

The practice of using combined numerical-experimental methods has demonstrated their effectiveness in solving various problems, including those related to structural vibrations. Similar methodologies are based on conducting experimental and numerical studies, comparing them, and subsequently refining numerical models under different values of significant factors^25,35,38,40^. This allows obtaining adequate (in relation to experiments) regularities of the criterion changes based on the significant factors of the process.

Existing methods for determining the natural oscillations of surfaces with holes^8,18,19^ or separately arranged volumetric riffles^39^ cannot be applied to combined perforated-riffled surfaces due to insufficient accuracy of the results. The complexity lies in the specific of the surface design, which is structural and consists of a group of holes and riffles that are translated in two coordinates. The need to determine the combined influence of the hole and riffle parameters creates a scientific problem and, at the same time, a practical need for relevant data.

The scientific novelty of the work is the development of a methodology for identifying the natural oscillation frequencies of perforated riffled surfaces, which allows determining the influence of hole and volumetric riffles parameters with sufficient accuracy and to study resonance phenomena under variable vibration conditions.

Thus, the goal of the study is to investigate the natural oscillation frequency of the riffled PVS using a combined experimental–numerical method with justification of rational values of parameters.

## Material and methods

### Methods

The research methodology consisted of the following stages: creation of physical PVS specimens; experimental identification of the natural oscillation frequency; numerical modeling using FEM; verification of the adequacy of numerical models (Abaqus_CAE version 2017) by comparing them with the results of experimental studies; calculation in the obtained adequate numerical models of variations in significant parameters of PVS; analysis of the results and obtaining the regularities of variation of the natural oscillation frequency of the riffled PVS from its parameters.

#### *Experimental identification*

At the first stage, experimental studies of PVS (Fig. 1) were carried out: basic (non-riffled) with elongated holes; riffled with volumetric stamped riffles and holes. To increase the accuracy of the research, samples of basic and designed PVS with two sizes of holes and riffles were produced. A rigid frame was manufactured to restrain displacements and rotation of the PVS in the rigid sections.

**Fig. 2:**
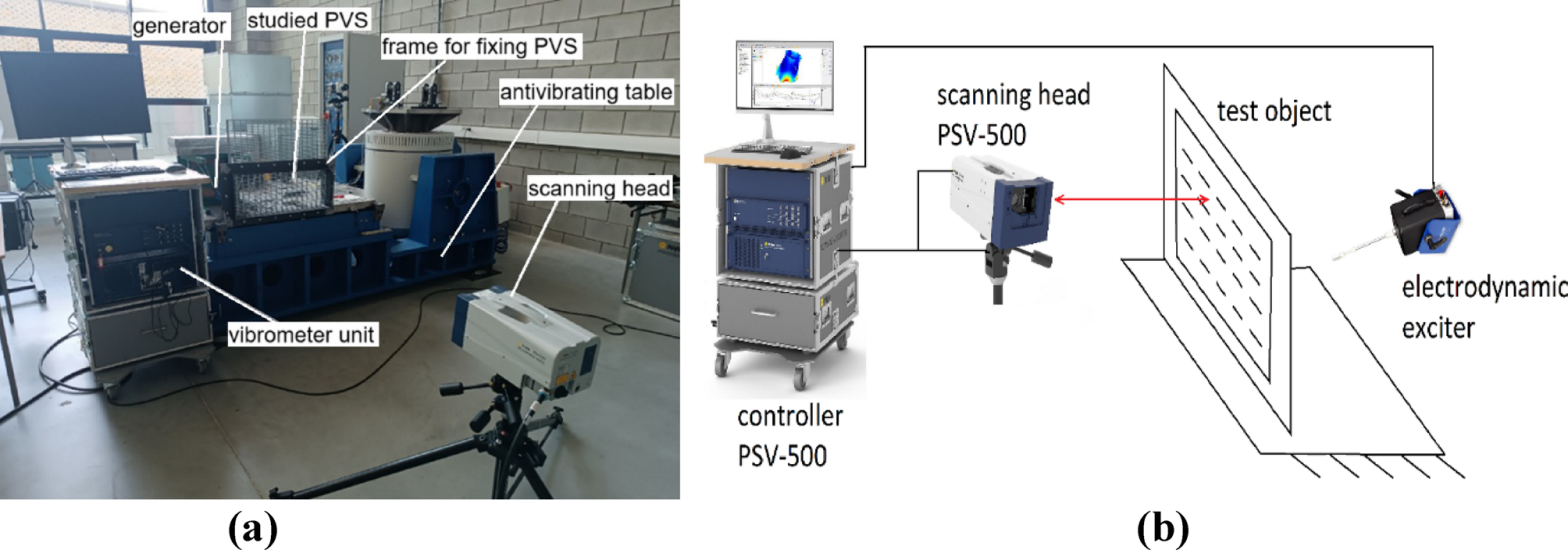
Experimental identification of the natural oscillation frequencies of plates. (**a**) View of the measurement process; (**b**) connection scheme.

For experimental identification of the natural frequencies, a testing bench was used (see Fig. 2), which consisted of: PSV-500 scanning vibrometer, clamping frame (item 2) for fixing samples of PVS (item 1), electromechanical shaker (item 3) for small amplitude excitation, contactless scanning head model PSV-I-500 (item 5) for laser measurement of oscillations and clamping frame with embedded specimen is fixed on the antivibrating table (item 6).

**Fig. 3:**
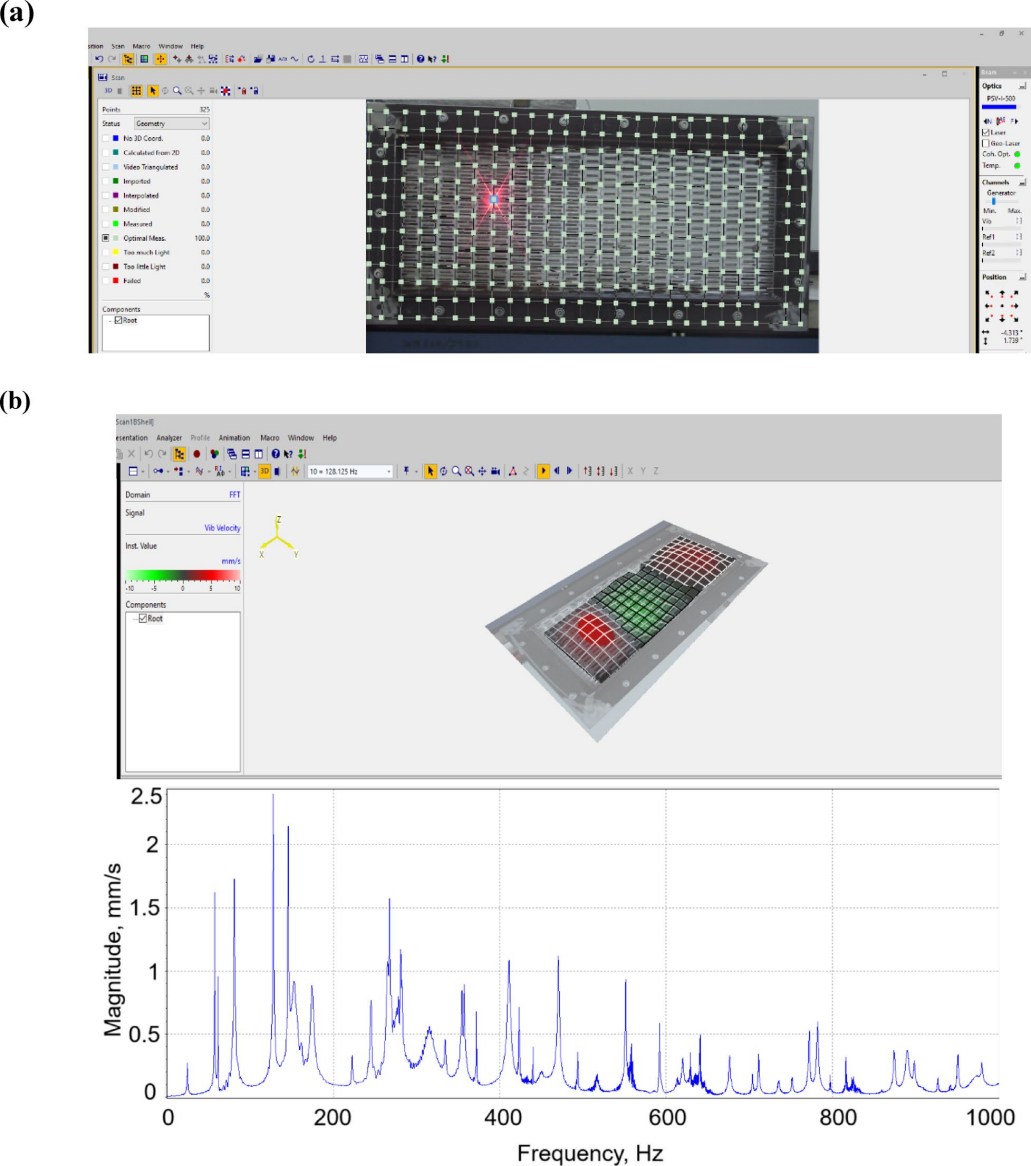
PSV-500 software. (**a**) Setting conditions; (**b**) processing experimental data.

The experiments were carried out in the following sequence. Firstly the investigated PVS samples were installed in the frame. Next, the point shaker was glued to the plate, followed by installation of the scanning head. Then, scanning points and measurement parameters were configured in the PSV-500 controller (Fig. 3a). The experiment was conducted at each predefined point. Finally, the results were visualized in controller PSV-500 (Fig. 3b).

: Research conditions set in the vibrometer included the number of points, repetition and measurement boundaries. For accuracy of measurements the grid with the number of points 13 × 25 (Fig. 3a) was set. Additional measurements with a larger number of points (26 × 50) showed not significant change of values of the natural oscillations of PVS (up to 0.04–0.1%).

The conducted analysis of the oscillation frequency in PVS separation equipment, both commercially produced and within the scope of scientific research, allowed for the identification of the range of variation, specifically 3–50 Hz^41,42^.

To extend the application range of the research results, 6 fundamental modes of oscillation (Table 1) were analyzed with the following half-waves (x,y), where x corresponds to the long side of the plate, y corresponds to the short side.

**Table 1:** Types of surface oscillations.

Mode №	Number of half-waves (x,y)	Visualization of the oscillation type	Mode no	Number of half-waves (x,y)	Visualization of the oscillation type
1	(1,1)		4	(4,2)	
2	(2,1)		5	(4,3)	
3	(3,1)		6	(4,4)	

#### Numerical modeling

**Fig. 4:**
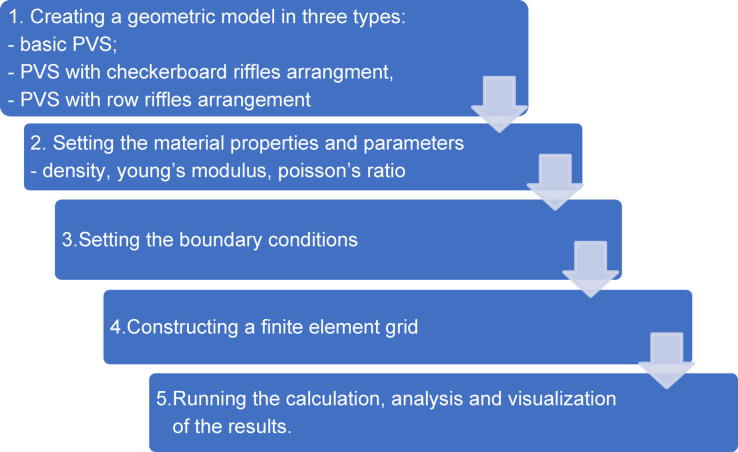
Algorithm of finite element simulations in Abaqus_CAE.

**Fig. 5:**
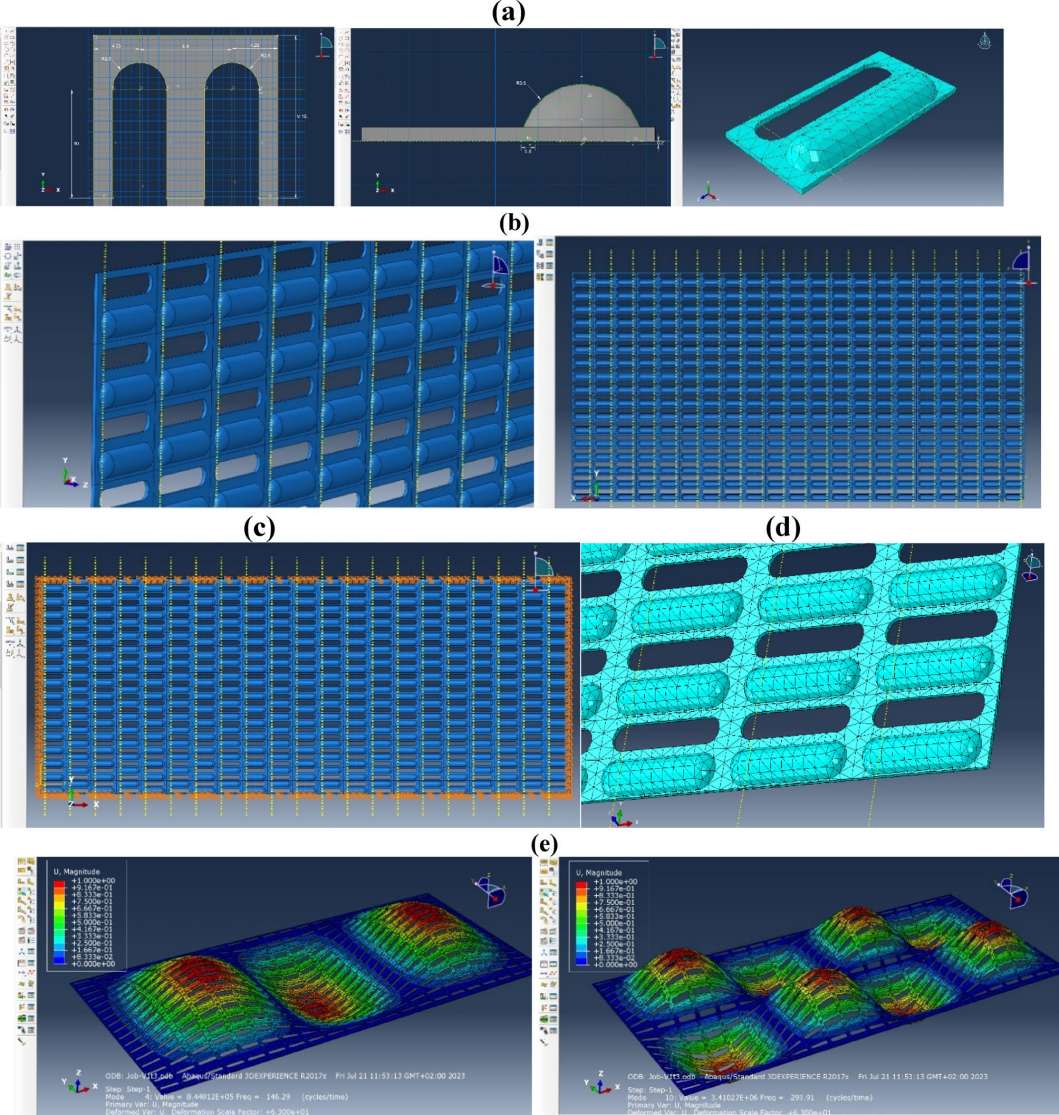
Selected steps of modeling PVS oscillations in Abaqus_CAE. (**a**) construction of the geometry of the elementary part; (**b**) construction of the geometric model of the entire PVS; (**c**) introducing boundary conditions; (**d**) creation of a grid with specified elements; (**e**) visualization of the results.

Abaqus_CAE by SIMULIA software with the following algorithm (Figs. 4 and 5) was used for numerical simulation of FEM.

For the studies in Abaqus_CAE, the following conditions characterizing the field of research have been adopted (Table 2).

**Table 2:** Mechanical and numerical properties of the finite element model.

Indicators	Values
Type the model	the linear model of elastic isotropic material
Geometry construction type	translation of parts (part) and their bonding (tie)
Boundary condition	displacement/rotation – rigid fixing of edges
Density, g/cm^3^	7.85
Young’s modulus, GPa	210
Poisson’s ratio	0.3
Type finite element mesh	quadratic hexahedral C3D20R / tetrahedral C3D10
Size of the finite element	2.6–2.8 mm
Total number of model elements	236,880

During the research, we checked the influence of mesh parameters on the reliability of the data. Numerical studies were carried out with the main mesh parameters (Table 2): total number of model elements 236–250 thousand units; element type – quadratic tetrahedral elements of type C3D10; approximate global size 2.6–2.8; maximum deviation factor 0.1. For the adopted number of finite elements of 248 thousand, their increasing to 1147 thousand leads to a change in the values of natural frequencies of oscillations of no more than 3.3%. The practical application of the proposed methodology is accompanied by the fact that increasing the number of elements significantly (by 262%) simultaneously increases the calculation time. Moreover, the conducted experiments confirmed the adequacy of the FE-model data. 

For the study, one of the most common schemes for fixing perforated surfaces was chosen, which is used for modal analysis—rigid fixation of all four sides (CCCC). For rigid fixation of the perforated surface during experiments, a frame with bolted clamps was developed (Fig. 2). In FE-models, CCCC fixing is performed, namely: type displacement/rotation are constrained.

#### Verification of the adequacy of numerical models

**Fig. 6:**
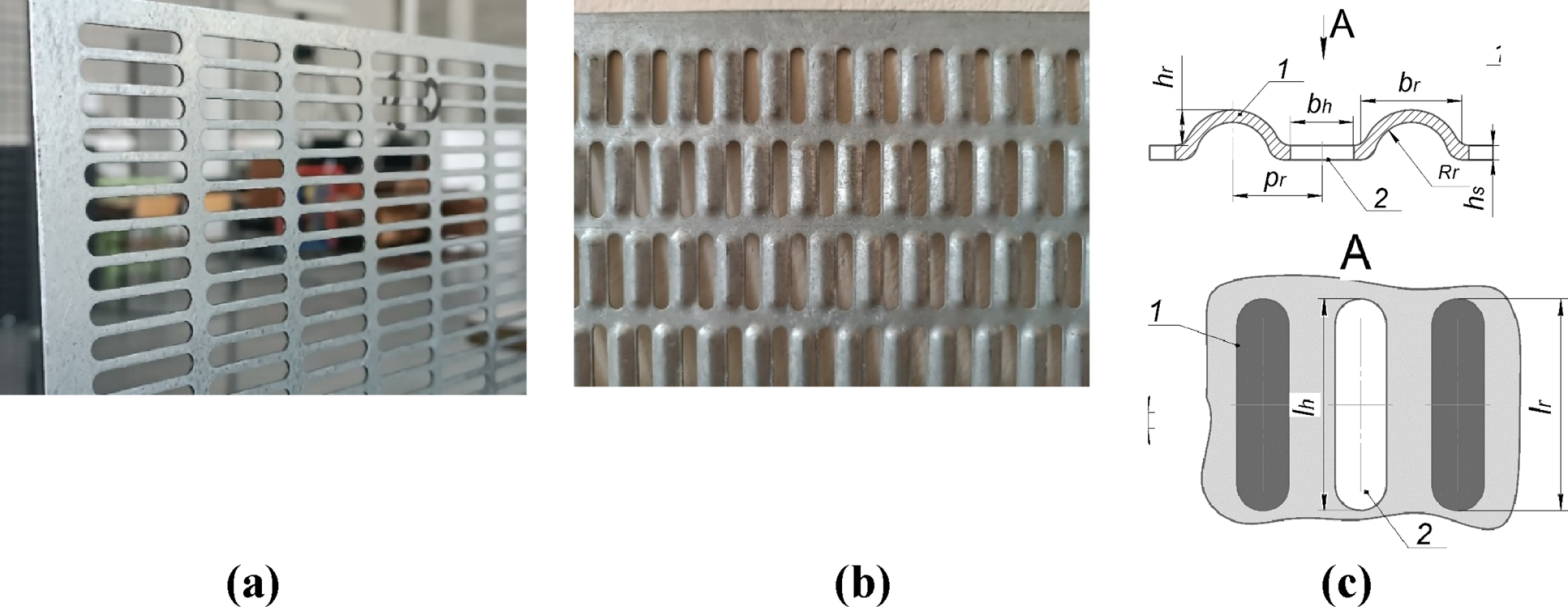
Riffled perforated vibration surfaces. (**a**) Basic; (**b**) with riffles; (**c**) dimensions of one unit.

The next stage involved verifying the adequacy of the obtained numerical models by comparing their results with experimental data. The comparative analysis was conducted for similar (experimental and numerical model) modes of oscillation, determining the relative error in the results. Further calculations were performed in adequate numerical models by varying the design significant parameters of basic and riffled PVS: plate thickness *h*_*s*_, riffle height *h*_*r*_ (radius *R*_*r*_) and bonding efficiency as pitch of holes or riffles *p*_*r*_. For more details we refer to Figs. 5a and 6c.

Statistical processing of the results allowed to obtain response surfaces (dependences) of the natural oscillation frequency of different types of PVS on significant factors. Also, regression equations characterizing changes of criterion (the natural frequency of the plate) from factors (parameters of PVS) involving their level of influence were obtained.

### Equipment and materials

Samples of basic PVS with elongated holes arranged in rows (Fig. 6a) and developed riffled PVS (Fig. 6b), with the following characteristics (Table 3).

**Table 3:** Parameters of investigated PVS samples.

Parameters	Indicators	Basic PVS with elongated holes		Riffled PVS	
		No 1	No 2	No 3	No 4
Construction					
PVS dimensions, mm: length/width/thickness	*l*_*s*_/*b*_*s*_/*h*_*s*_	630/240/1			
Hole dimensions, mm: width/length	*b*_*h*_/*l*_*h*_	3.2/25	5/25	3.2/25	5/25
Hole pitch arrangement, mm: longitudinal/transverse	*p*_*l*_/*p*_*r*_	30/12	30/15	30/12	30/15
Number of holes, pcs	*n*_*h*_	798	630	399	315
Riffle dimensions, mm: length/width	*l*_*r*_ / *b*_*r*_	–	−	18/3.2	25/5
Number of riffles, pcs	*n*_*r*_	–	−	399	315
Material					
Still		S235 JR (EN 10,025–2)			
Density, g/cm^3^	*Ρ*	7.847			
Young’s modulus, GPa	*E*	210			
Yield strength, MPa	*σ*_*y*_	207			
Tensile strength, MPa	*σ*_*t*_	345			

**Fig. 7:**
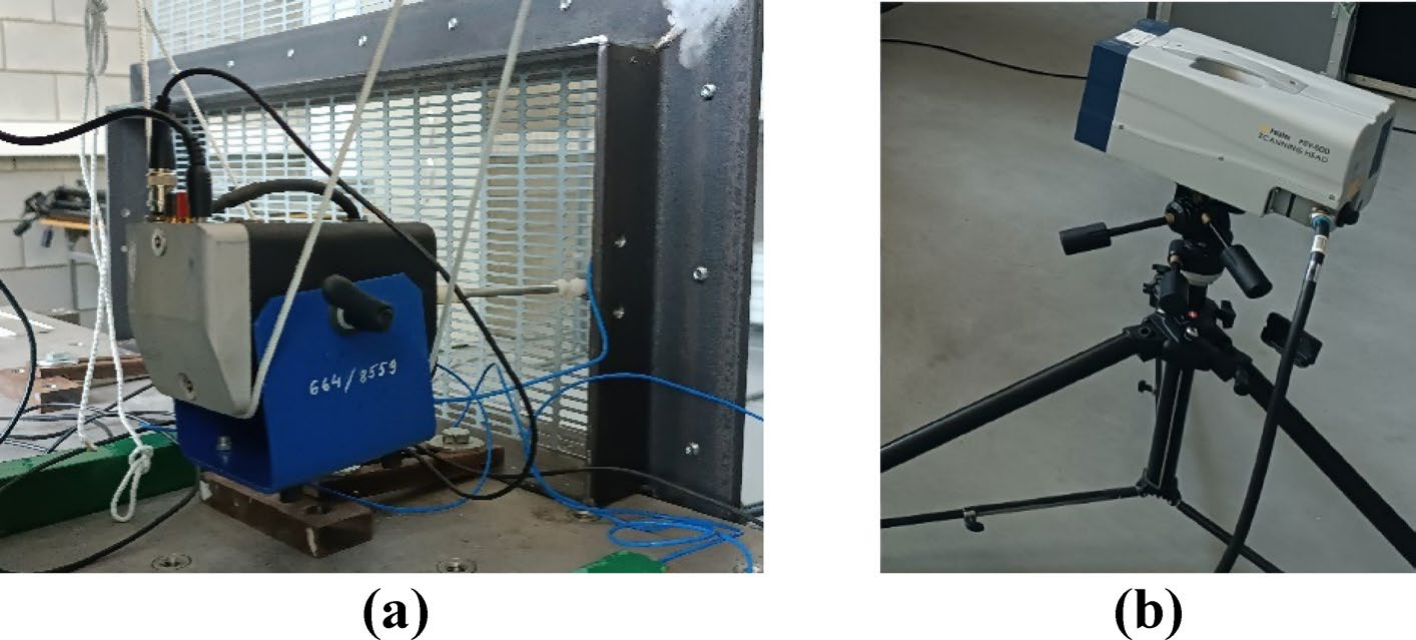
Equipment for experiments. (**a**) Generator K2004E01 Kit; (**b**) scanning head model PSV-I-500, version GEO CO.

Dedicated equipment was used for experimental studies: an oscillation generator, vibrometer, scanning head (Fig. 7; Tables 4, 5).

**Table 4:** Parameters of vibrometer PSV-500.

Characteristic	Value
Measuring range of vibration velocity, mm/s	0…10,000
Operating frequency ranges, kHz	0…100
Relative error of vibration velocity at 0…20 kHz, %	± 1
Wavelength of measuring laser, nm	633 (red)
Laser power, not exceeding, mW	1

**Table 5:** Oscillation generator parameters K2004E01 Kit.

Characteristic	Value
Force rating, lbf (N)	7 (31)
Max frequency, Hz	9 000
Max stroke, in (mm)	0.5 (13)
Amplifier model	Integrated

The Abaqus CAE 2017 by SIMULIA software was used for numerical modeling of FEM. MATLAB and Grapher 10 was used to process and analyze the results.

## Calculation and results

### Verification of the adequacy of the obtained numerical models

In order to be able to widely use FEM to solve the problems, comparative studies with experiments have been carried out. As a result of numerical modeling (performed in Abaqus) and experimental studies on the PSV-500 vibrometer, following the described methodology of Section "Material and methods", natural frequencies were obtained for basic and riffled PVS for 6 fundamental modes of vibrations as well (Table 6).

**Table 6:** Visual images of surface oscillations by various methods of study.

Mode	Experiment	FEM
No 1		
No 2		
No 3		
No 4		
No 5		
No 6		

The comparative results numerical and experimental studies are summarized in Table 7. The relative error of the results was determined by the formula: *Δ* = *(ω*_*ab*_*-ω*_*ex*_*)/ ω*_*ab*_ 100%, where *ω*_*ab*_*, ω*_*ex*_ – the natural oscillation frequencies of PVS, obtained numerically in Abaqus/CAE and experimentally, respectively.

**Table 7:** The natural oscillation frequency of PVS.

Indicators	Mode					
	No 1	No 2	No 3	No 4	No 5	No 6
PVS with riffles in checkerboard arrangement (*b*_*h*_ = 5 mm)						
*ω*_*ab*_, Hz	60.78	90.31	139.49	294.35	429.99	610.84
*ω*_*ex*,_ Hz	62.44	88.37	135.93	284.69	443.44	600.31
*Δ*, %	2.73	2.14	2.55	3.28	3.13	1.72
PVS with riffles in checkerboard arrangement (*b*_*h*_ = 3.2 mm)						
*ω*_*ab*_, Hz	58.98	81.59	120.82	263.24	401.05	588.38
*ω*_*ex*,_ Hz	56.64	78.5	122.19	258.43	411.56	566.28
*Δ*, %	3.96	3.78	1.13	1.82	2.62	3.75
Base PVS (*b*_*h*_ = 5 mm)						
*ω*_*ab*_, Hz	53.87	66.88	89.52	210.03	342.2	516.76
*ω*_*ex*,_ Hz	55.38	68.625	86.38	206.88	336.25	499.93
*Δ*, %	2.80	2.60	3.50	1.5	1.73	3.25
Base PVS (*b*_*h*_ = 3.2 mm)						
*ω*_*ab*_, Hz	46.95	61.65	86.68	197.07	310.04	459.65
*ω*_*ex*,_ Hz	45.36	59.43	84.43	197.81	304.9	443.15
*Δ*, %	3.38	3.60	2.59	0.37	1.65	3.58

Based on the conducted studies and results analysis, the following conclusions were drawn:

- Adequacy of numerical models in relation to experimental data is confirmed by insignificant relative error of 0.37–3.96%;
- Numerical models can be used for advanced simulations of perforated and riffled structures;
- A significant difference between the natural oscillation frequency of basic perforated plates and developed riffled plates amounted to: 11.3–35% at holes with *b*_*h*_ = 5 mm and 20.39–28.25% at holes with *b*_*h*_ = 3.2 mm, which requires detailed elaboration of new methods and obtaining dependences on the parameters of riffles.

Increasing the size of the riffles and the width of the jumpers between the holes leads to the surface approaching a flat shape. The flat shape of the surface is better adapted to FE-modeling algorithms, particularly in the successful arrangement of elements. This results in higher accuracy in numerical calculations and minimal deviation from experimental data.

### Modeling of the natural oscillation frequency of perforated riffled vibration surfaces

Considering the proven adequacy of numerical models (paragraph 4.1), the analysis according to the criterion of the natural oscillation frequency of PVS with the variation of significant factors will be conducted. Preliminary experiments and their analysis identified the following significant factors: geometrical parameters of riffles and holes, number and pitch riffles arrangement and holes, material thickness and properties of PVS, overall dimensions of PVS.

At the same time, selected parameters are optimal in relation to the process of sifting particles of loose materials through the holes. Thus, the riffle length *l*_*r*_, in terms of maximum orientation of particles into the holes, should correspond to the hole length *l*_*h*_. The riffle height *h*_*r*_ is determined by the value of the punch radius *R*_*r*_ (tool of the mechanical press), that is limited by the material properties and PVS thickness *h*_*s*_. It should be noted that decreasing the riffle height leads to a decrease in the orientation effect of loose material components when they are sifted through PVS holes. Regarding the shape and size of most loose material components, we have a correction factor between width and thickness *k*_*par*_ = 0.5–0.7. The studied PVS have riffles with a rounded cross-sectional shape, which makes it possible to use the riffle radius *R*_*r*_ = *k*_*par*_ × *b*_*h*_ instead of the height of *h*_*r*_. To narrow down the scope of present study, we take the following range of riffle radius variation: at *b*_*h*_ = 3.2 mm *R*_*r*_ = 1.8–2.4 mm; at *b*_*h*_ = 5 mm *R*_*r*_ = 2.5–3.5 mm.

Ranges of variation of partition width and surface thickness PVS are selected from the conditions of economic costs minimization, maximization the technological sifting of loose medium particles and the results of known studies^13,38,43,44^. The ranges of variation in PVS thickness were *h*_*s*_ = 0.8–1.2 mm; pitch of riffles arrangement at *b*_*h*_ = 3.2 mm *p*_*b*_ = 6–6.8 mm; at *b*_*h*_ = 5 mm *p*_*b*_ = 7.5–8.5 mm.

Studies were conducted for the remaining factors with the following levels of variation (Tables 8, 9).

**Table 8:** Ranges of selected factors for riffled PVS.

Level and range of factors variation	Factors				
	*X*_1_	*X*_2_		*X*_3_	
	Surface thickness *h*_*s*_, mm	Riffle radius *R*_*r*_, mm		Pitch of riffles arrangement *p*_*b*_, mm	
		*b*_*h*_ = 3.2 mm	*b*_*h*_ = 5 mm	*b*_*h*_ = 3.2 mm	*b*_*h*_ = 5 mm
+ 1	1.2	1.8	2.5	6	7.5
0	1	2.1	3	6.4	8
− 1	0.8	2.4	3.5	6.8	8.5

**Table 9:** Ranges of selected factors for PVS without riffles.

Level and range of factors variation	Factors		
	*X*_1_	*X*_2_	
	Surface thickness *h*_*s*_, mm	Pitch of holes arrangement p_h_, mm	
		*b*_*h*_ = 3.2 mm	*b*_*h*_ = 5 mm
+ 1	1.2	6	7.5
0	1	6.4	8
− 1	0.8	6.8	8.5

**Fig. 8:**
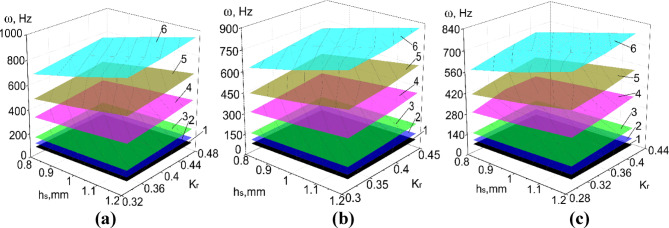
The natural frequency of PVS with checkerboard riffles arrangement. (**a**) *p*_b_ = 7.5 mm; (**b**) *p*_b_ = 8 mm; (**c**) *p*_b_ = 8.5 mm. (hole width b_h_ = 5 mm; p_l_ = 30 mm; n_rb_ = 16 pcs.; n_rl_ = 21 pcs.; l = 0.2 mm; 1–6 modes of oscillations).

**Fig. 9:**
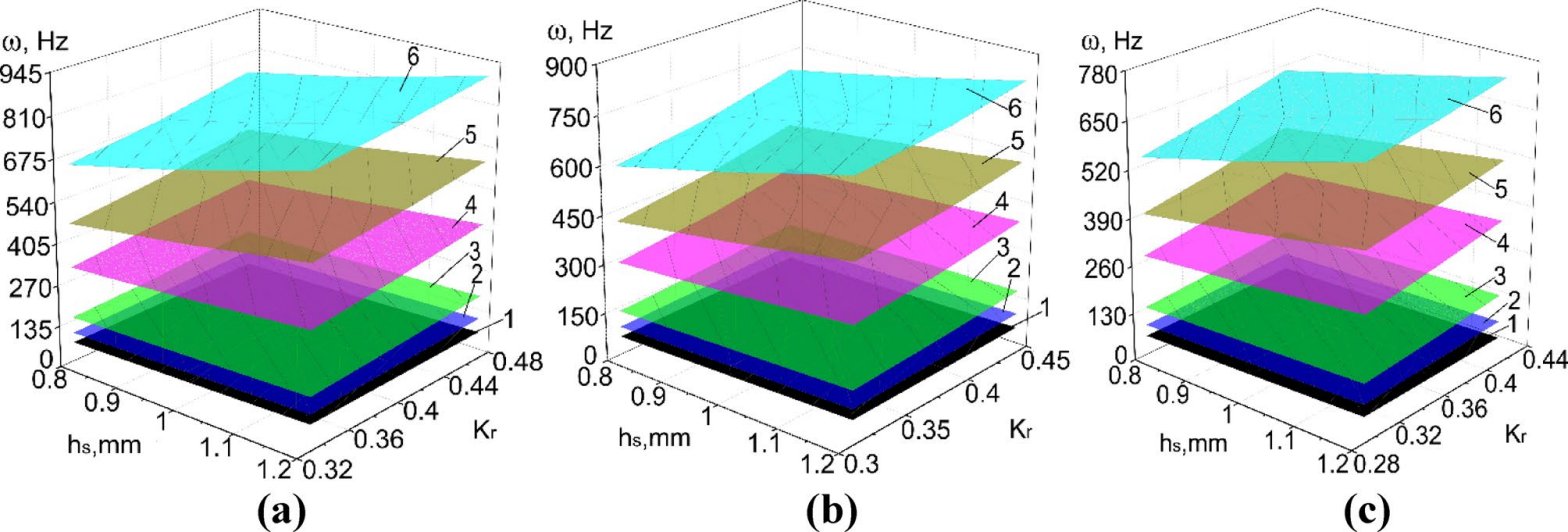
The natural oscillation frequency of PVS with row riffles arrangement. (**a**) *p*_b_ = 7.5 mm; (**b**) *p*_b_ = 8 mm; (**c**) *p*_b_ = 8.5 mm. (hole width b_h_ = 5 mm; p_l_ = 30 mm, n_rb_ = 16 pcs.; n_rl_ = 21 pcs.; l = 0.2 mm; 1–6 modes of oscillations).

**Fig. 10:**
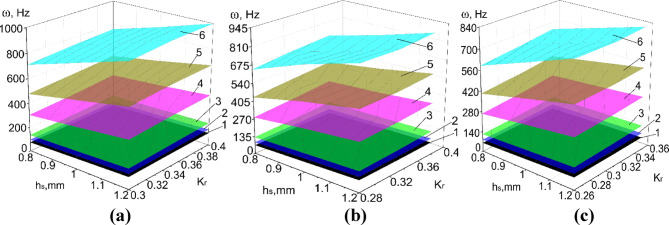
The natural oscillation frequency of PVS with checkerboard riffles arrangement. (**a**) *p*_b_ = 6 mm; (**b**) *p*_b_ = 6.4 mm; (**c**) *p*_b_ = 6.8 mm. (hole width b_h_ = 3.2 mm; p_l_ = 30 mm; n_rb_ = 19 pcs.; n_rl_ = 21 pcs.; l = 0.1 mm; 1–6 modes of oscillations).

**Fig. 11:**
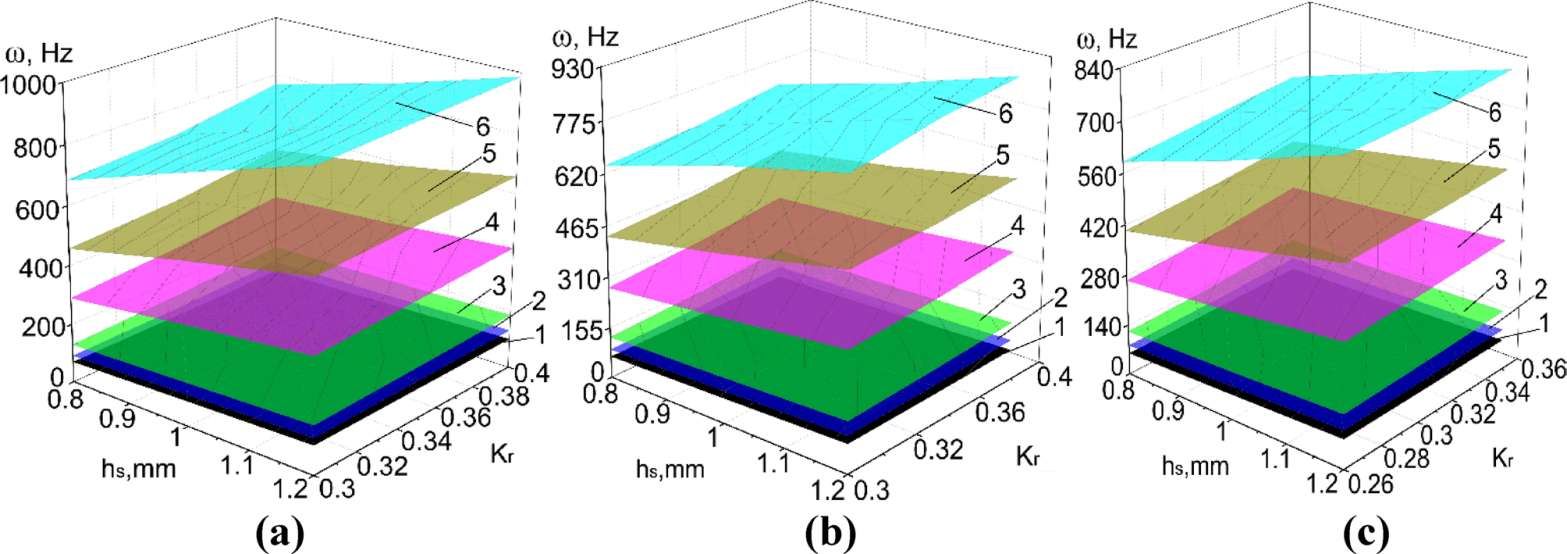
The natural oscillation frequency of PVS with row riffles arrangement. (**a**) *p*_b_ = 6 mm; (**b**) *p*_b_ = 6.4 mm; (**c**) *p*_b_ = 6.8 mm. (hole width b_h_ = 3.2 mm; p_l_ = 30 mm; n_rb_ = 19 pcs.; n_rl_ = 21 pcs.; l = 0.1 mm; 1–6 modes of oscillations).

**Fig. 12:**
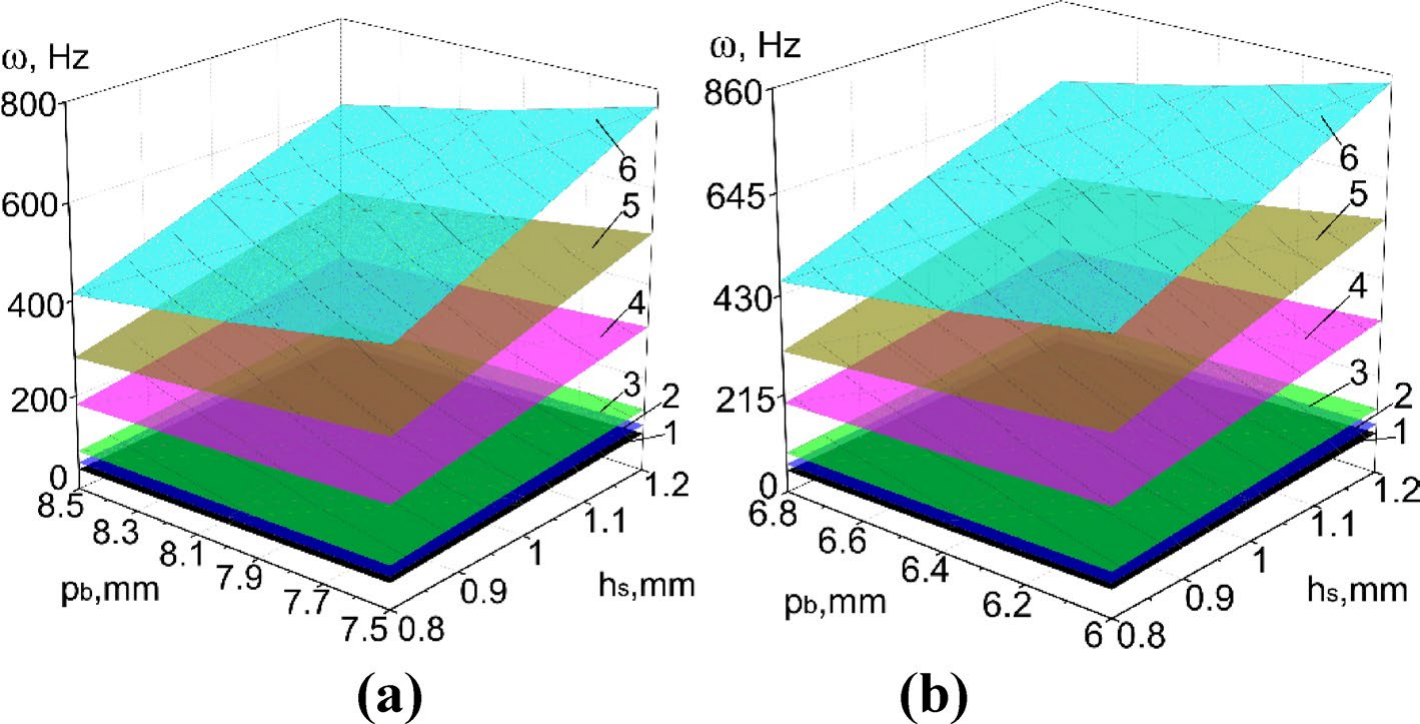
The natural oscillation frequency of PVS without riffles. (**a**) Hole width *b*_*h*_ = 5 mm (*p*_*l*_ = 30 mm; *n*_*rb*_ = 32 pcs.; *n*_*rl*_ = 21 pcs.; *l* = 0 mm); (**b**) hole width *b*_*h*_ = 3.2 mm (*p*_*l*_ = 30 mm; *n*_*rb*_ = 38 pcs.; *n*_*rl*_ = 21 pcs.; *l* = 0 mm; 1–6 modes of oscillations).

Results of numerical calculations of PVS in Abaqus CAE_2017, taking into account ranges of significant factors (Tables 8, 9) are presented in Figs. 8, 9, 10, 11 and 12.

Considering the ranges of variation of the forced oscillation frequency of PVS in the technological processes of sifting loose materials, we focus on the first oscillation mode. We will analyze the obtained results.

To facilitate data analysis, a relative parameter will be used to characterize the level of corrugation of the perforated surface – the corrugation coefficient Kr. This coefficient is defined as the ratio of the riffle radius *R*r to the pitch of riffle arrangement *p*_*b*_:

1 $${\text{K}}_{r}={R}_{r}/{p}_{\text{b}}.$$

Based on the experimental parameters adopted (Tables 8, 9), the perforated surfaces have the following variation range of the coefficient ${\text{K}}_{r}$=0.26–0.46. Another significant factor that characterizes the structural stiffness is the surface thickness. Thus, the obtained dependencies of the natural oscillation frequency of PSV on its thickness, hole sizes, and the ${\text{K}}_{r}$ coefficient (Figs. 8, 9, 10, 11, 12).

The statistical data processing (for 1 mode of oscillation) was conducted using a central composite uniform-rotatable design of the second order. As a result of data processing, a regression equation for riffled PVS with checkerboard riffles arrangement was obtained, taking into account significant factors (Table 8) and significant regression coefficients:

2 $$\begin{aligned} Y & = 74.93 \, + \, 9.9396_{X1} + 3.7912_{X2} - 6.4125_{X3} \\ & \quad - 0.1575 -_{X12} - 1.1533_{X13} + 0.095X_{3}^{2} . \\ \end{aligned}$$

The regression equation shows that the most influential on the response function (the natural frequency of PVS) is the factor $X_{1}$ – surface thickness *h*_*s*_ (the measure of influence is 9.94), with an increase in which the frequency value increases. The second most influential factor is $X_{3}$ – pitch of riffles arrangement *p*_*b*_ (the degree of influence is 6.41), an increase in which leads to a decrease in the natural oscillation frequency of PVS (as evidenced by the "–" sign before the corresponding coefficient). The factor $X_{2}$ – riffle radius *R*_*r*_ is the least influential of these factors (the measure of influence is 3.79). Interactions of factors have a much smaller impact on the response function.

The conclusion regarding the significance of factors was made by checking using the Student’s t-test. The adequacy of the obtained polynomial was verified using the Fisher’s criterion: $F_{es} = 1.85 \le F_{{\left( {0,05;f_{{}} f_{y} } \right)}}$=2.12.

The calculated value of the optimization criterion is less than the tabulated value of the F-criterion at a 5% significance level. Therefore, the hypothesis of the adequacy of Eq. (2) can be considered true with a 95% probability.

A similar regression equation was obtained for riffled PVS with a row riffles arrangement:

3 $$\begin{aligned} Y & = \, 68.06 \, + \, 9.7837_{X1} + \, 2.155 {-} \, 6.6513_{X3} {-} \, 0.4992_{X12} \\ & \quad - 1.2167_{X13} + 1.3658_{X23} + 0.511X_{1}^{2} + 0.8175X_{2}^{2} + 0.8456X_{3}^{2} \\ \end{aligned}$$

with $$F_{es} =$$ 1.01 $$\le F_{table} =$$ 2.12.

By analogy with the riffled PVS with checkerboard arrangement, the most influential factor is the factor $X_{1}$ – surface thickness *h*_*s*_ (the degree of influence is 9.78), with an increase in which the value of the natural oscillation frequency increases. The next most influential factor is $X_{3}$ – pitch of riffles arrangement *p*_*b*_ (the degree of influence is 6.65), an increase in which leads to a decrease in the natural oscillation frequency of PVS. The least influential is riffle radius *R*_*r*_ (the degree of influence is 2.15), an increase in which leads to an increase in the natural oscillation frequency of PVS. The interactions of these two factors have a much smaller effect on the response function.

The regression equation in decoded form has the following expression:

a) for PVS with checkerboard riffles arrangement

4 $$\omega = - 131.3856 + 148.237h_{s} + 9.3222R_{r} + 12.9154p_{b} - 1.575h_{s} R_{r} - 11.533h_{s} p_{b} + 0.38p_{b}^{2}$$

b) for PVS with row riffles arrangement

5 $$\begin{aligned} \omega & = 249.2149 \, + \, 135.6805h_{s} {-} \, 54.0236R_{r} {-} \, 54.031p_{b} {-} \, 4.992h_{s} R_{r} \\ & \quad {-} \, 12.167h_{s} p_{b} + \, 5.4632R_{r} p_{b} + \, 12.775 + \, 3.27 + \, 3.3824p_{b}^{2} . \\ \end{aligned}$$

**Fig. 13:**
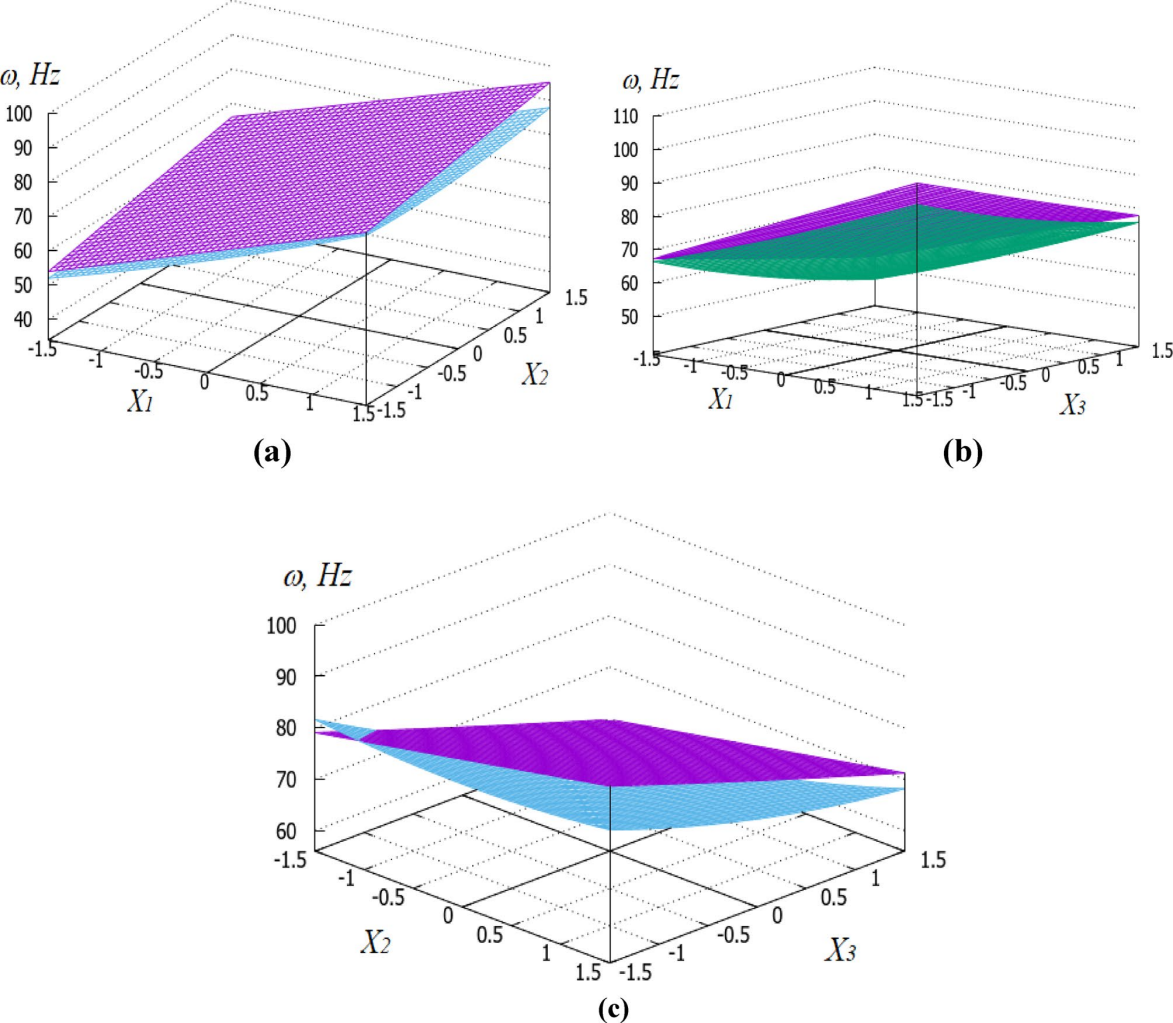
Dependencies of variation of the natural frequency (Hz) of riffled PVS depending on its: (**a**) thickness h_s_ (X_1_), (**b**) riffles’ radius R_r_ (X_2_); (**c**) pitch of their arrangement p_b_ (X_3_). Purple surface represents PVS with checkerboard riffles arrangement while green surface corresponds to PVS with row riffles arrangement.

A graphical representation of the patterns of changes in the natural oscillation frequency of riffled PVS with checkerboard and row riffles arrangement from significant factors is shown in Fig. 13.

The analysis of the dependencies shows that riffled PVS with row riffles arrangement have lower values of the natural oscillation frequency compared to PVS with checkerboard riffles arrangement. This is due to the formation of additional surface stiffness.

Statistical processing of the research data on the basic PVS with the factors (Table 9) allowed us to obtain the following regression equation:

6 $$Y = 60.7025 + \, 10.5475_{X1} - 8.5475_{X2} - 0.3325_{X1X2}$$

By analogy with riffled PVS, the most influential factor is $X_{1}$ – surface thickness *h*_*s*_ (the degree of influence is 10.54), with an increase in which the natural oscillation frequency increases. The less influential factor is $X_{2}$ – pitch hole arrangement *p*_*h*_ (the degree of influence is 8.55), an increase in which leads to a decrease in the natural oscillation frequency of PVS. The interactions of these two factors have a much smaller effect on the response function.

**Fig. 14:**
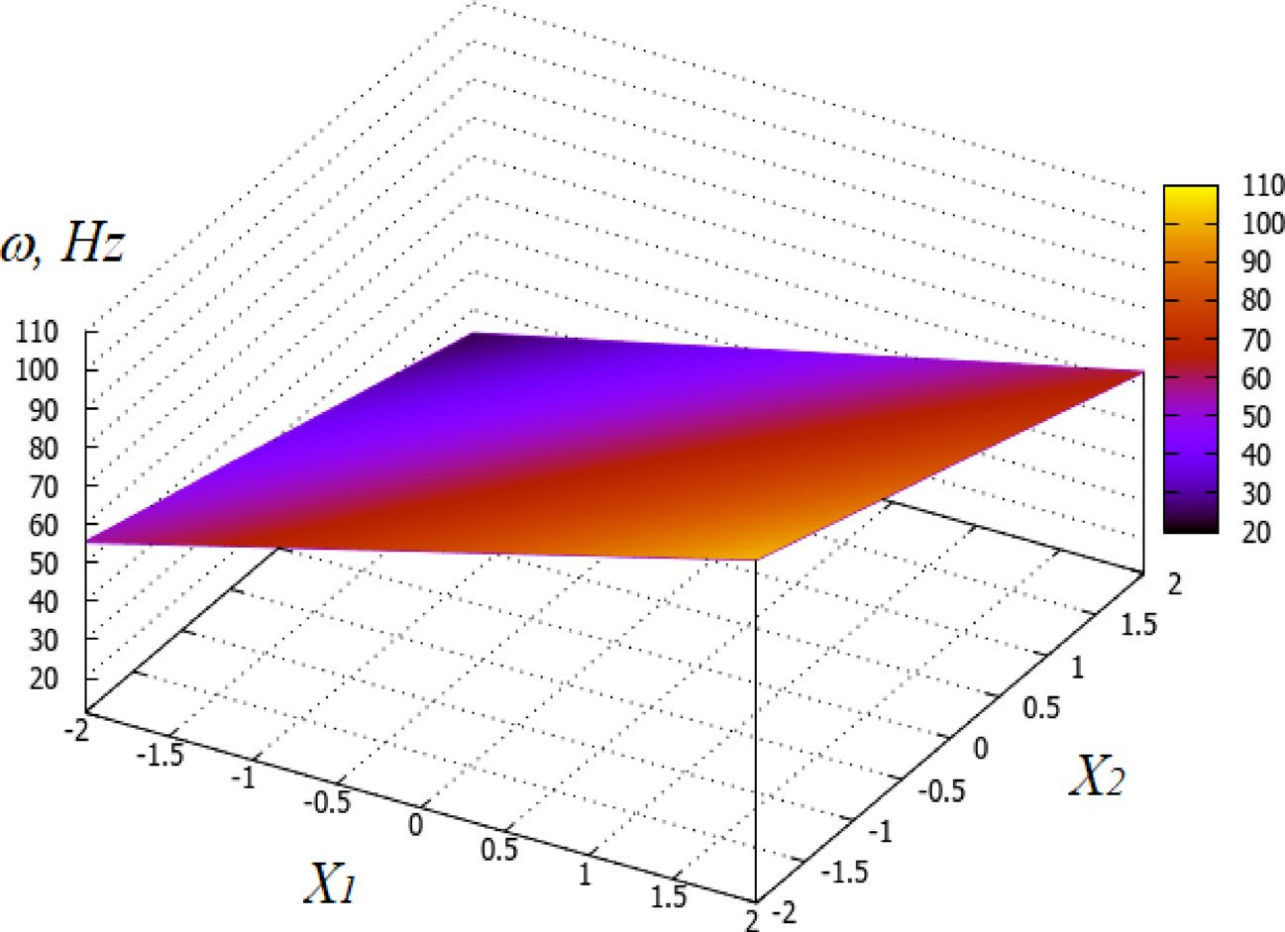
Dependence of the natural frequency (Hz) of the base PVS on its thickness h_s_ (X_1_) and the period of hole arrangement p_h_ (X_2_).

Dependencies of the natural oscillation frequency of the base PVS on the thickness (*X*_1_) and the period of hole arrangement (*X*_2_) are graphically presented in Fig. 14.

## Discussion

**Fig. 15:**
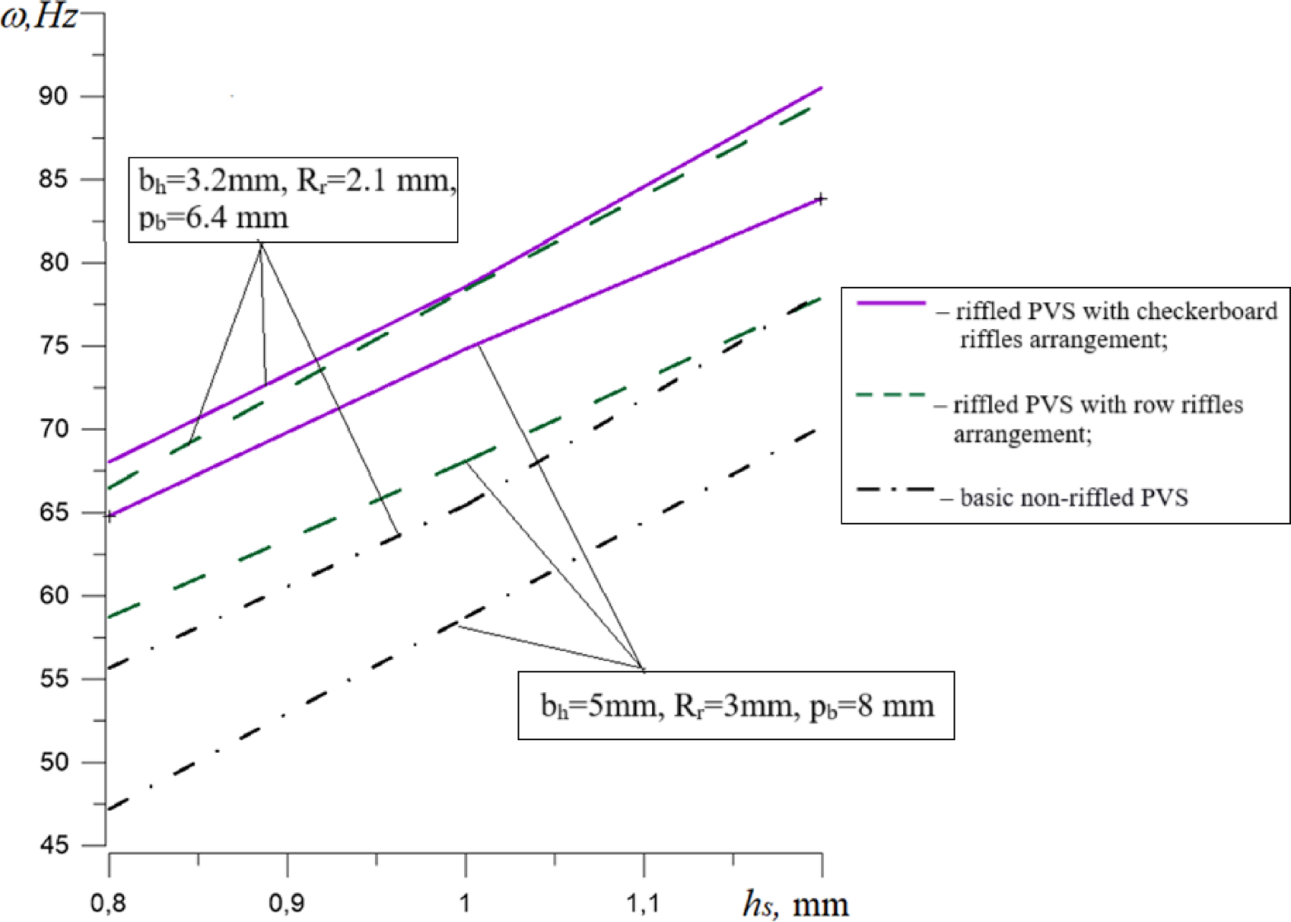
Dependencies of the natural frequencies of PVS on its thickness h_s._

**Fig. 16:**
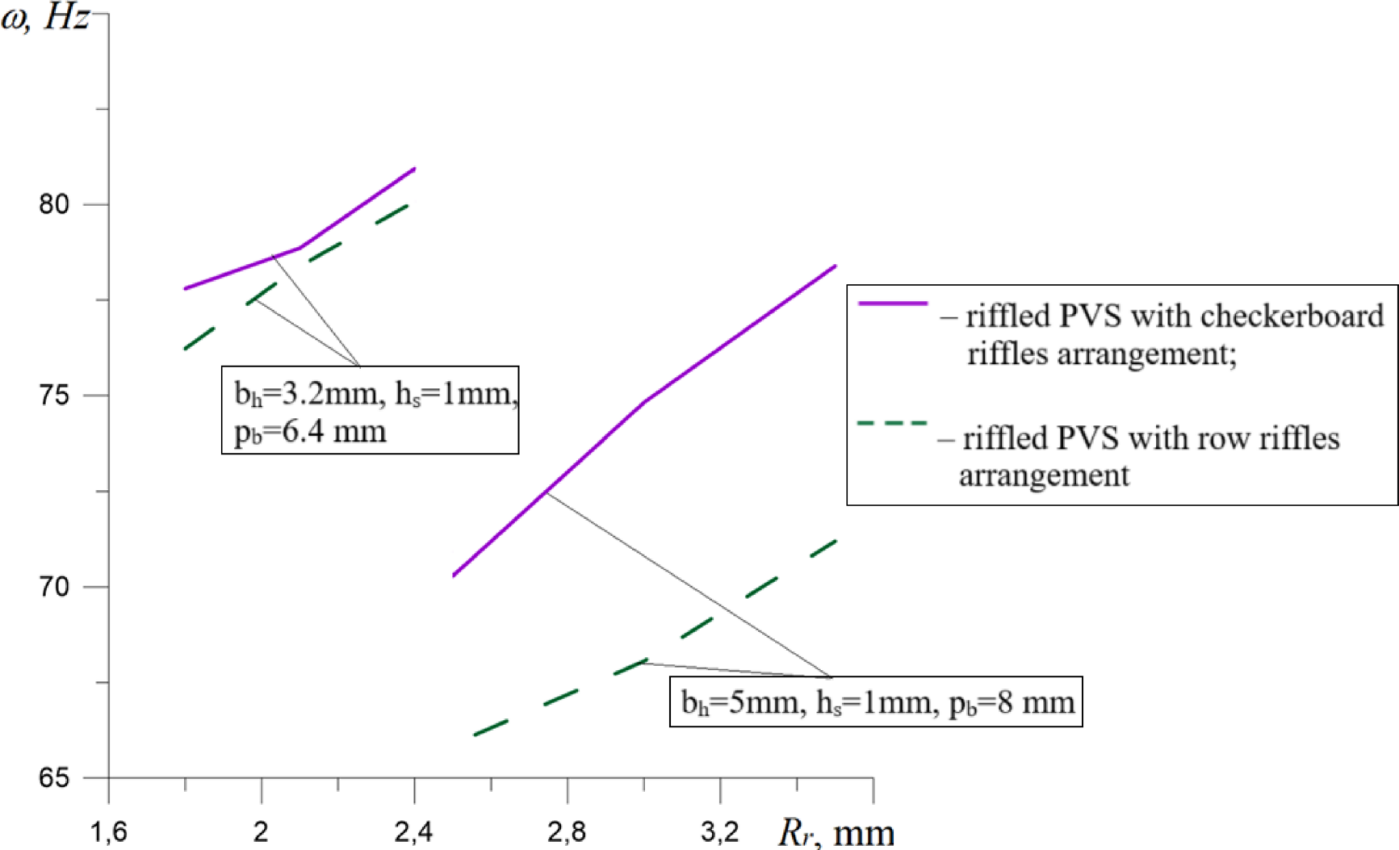
Dependencies of the natural frequencies of PVS on its riffles radius R_r._

**Fig. 17:**
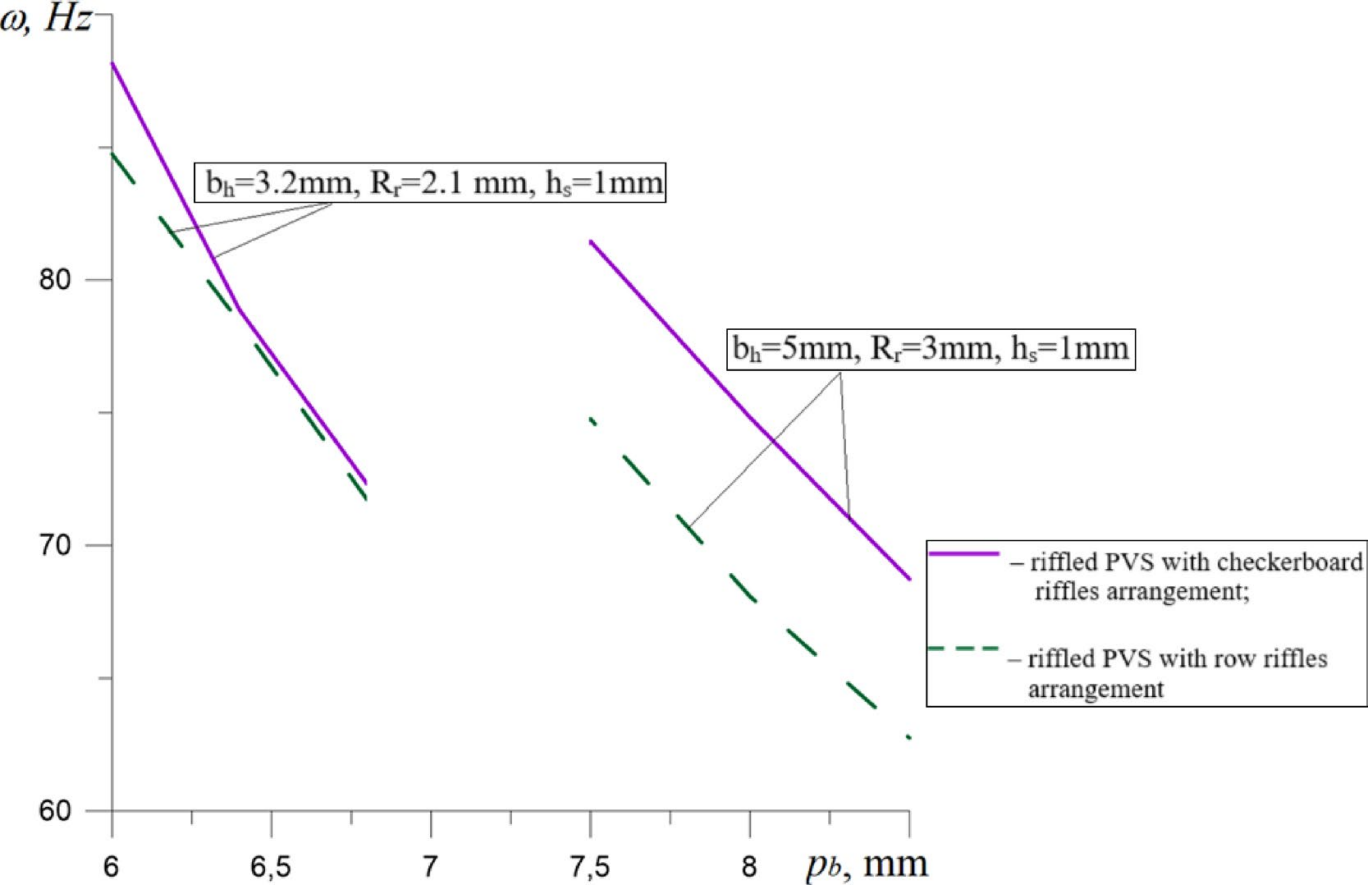
Dependences of the natural frequencies of PVS on the pitch of riffles arrangement p_b_.

To compare the influence of PVS design features on the change in their natural oscillation frequencies, dependencies were plotted (Figs. 15, 16, 17).

The analysis of the results (Fig. 15) revealed that an increase in PVS thickness for all experimental structures causes an increase in their natural oscillation frequencies by 29.39–48.71%. This is confirmed by the known analytical expressions for determining the natural oscillation frequencies of plates in which the plate thickness is present to ensure structural rigidity. The lowest natural oscillation frequency characteristic of basic non-riffled PVS is: 47.18–78.17 Hz.

The change in the PVS thickness, in the range studied, is the most significant factor influencing their natural oscillation frequency: for riffled PVS with checkerboard riffles arrangement increases by 29.4%/33.06% (*b*_*h*_ = 5 mm/ *b*_*h*_ = 3.2 mm); for riffled PVS with row riffles arrangement increases by 32.65%/34.78% (*b*_*h*_ = 5 mm/ *b*_*h*_ = 3.2 mm); for basic non-riffled PVS by 48.71%/40.42% (*b*_*h*_ = 5 mm/ *b*_*h*_ = 3.2 mm).

The type of PVS construction also affects the natural oscillation frequency. As a result of comparing the results of oscillation frequencies for different PVS designs, the following was established:

- PVS with checkerboard riffles arrangement in relation to the base PVS – decrease by 16.33–27.19% (*b*_*h*_ = 5 mm) and by13.63–18.16% (*b*_*h*_ = 3.2 mm);
- PVS with row riffles arrangement in relation to the base PVS – decrease by 9.95–19.67% (*b*_*h*_ = 5 mm) and by 12.78–16.51% (*b*_*h*_ = 3.2 mm);
- PVS with checkerboard riffles arrangement in relation to PVS with row riffles arrangement – decrease by 7.08–9.36% (*b*_*h*_ = 5 mm) and by 0.98–2.25% (*b*_*h*_ = 3.2 mm).

The analysis of the results (Fig. 16) revealed an increase in the natural frequencies of riffled PVS by 1.43–11.52% depending on the riffles radius R_r_. The use of row arrangement in comparison with checkerboard one causes a decrease in the values of the natural oscillation frequencies of PVS by 0.22–4.47% for small hole widths (*b*_*h*_ = 3.2 mm); 6.27–9.17% for larger hole widths (*b*_*h*_ = 5 mm).

The analysis of the results obtained (Fig. 17) shows a decrease in the natural frequencies of riffled PVS by 15.36–17.98% depending on the pitch riffles arrangement p_b_. In this case, the use of row riffles arrangement in comparison with checkerboard one causes a decrease in the values of the natural frequencies of PVS by: 0.6–3.88% with the hole width *b*_*h*_ = 3.2 mm; 8.21–9% – at width *b*_*h*_ = 5 mm.

## Summary

As a result of the research, a method for determining the natural frequencies of riffled PVS has been developed, which is based on numerical experimental methods. Adequate numerical models became the basis for substantiating the significance of the parameters of riffled PVS: surface thickness, radius of riffles, and their pitch arrangement.

The obtained ranges of variation of the natural frequencies of PVS were compared with the characteristic oscillation frequencies of the separation equipment, which narrowed the research area to the first mode of oscillation only.

The derived regression equations for the natural frequency of riffled PVS were used to determine the levels of the factors’ influence: the plate thickness, pitch arrangement, riffle height.

The dependences of changes in the natural frequency of riffled PVS with checkerboard and row arrangement of riffles and the basic non-riffled PVS were determined.

It has been found that the use of riffled PVS with checkerboard riffles arrangement provides the highest stiffness and consequently has the highest natural frequency.

The obtained regularities of the natural frequencies of riffled perforated plates will enable to predict the appearance of deformations in the form of cracks between the holes and to determine their technological durability.

## Electronic supplementary material

Below is the link to the electronic supplementary material.

Supplementary Material 1

## Data Availability

The datasets used and/or analysed during the current study available from the corresponding author on reasonable request.
