# Supplementary material for: Combined method of identification of free oscillations of perforated riffled plates
Source: Sci Rep. 2025 Jul 4;15:23992. doi: 10.1038/s41598-025-06614-5 (PMC12227721; doi:10.1038/s41598-025-06614-5)
Supplement: Supplementary file 1 — Supplementary Material 1 [file 41598_2025_6614_MOESM1_ESM.docx]

**APPENDIX**

| **Surface thickness**  ***h_s_*, mm** | **Riffle radius *R_r_*, mm** | **Pitch of riffles arrangement *p_b_*, mm** | **Natural frequency, Hz** | | | | | |
| --- | --- | --- | --- | --- | --- | --- | --- | --- |
|  |  |  | **Mode №1** | **Mode №2** | **Mode №3** | **Mode №4** | **Mode №5** | **Mode №6** |
| 0.8 | 2.5 | 7.5 | 67.08 | 97.94 | 149.41 | 319.16 | 471.22 | 673.48 |
| 1 | 2.5 | 7.5 | 77.71 | 108.04 | 160.52 | 347.67 | 526.4 | 766.96 |
| 1.2 | 2.5 | 7.5 | 89.34 | 120.02 | 173.67 | 383.73 | 593.52 | 875.12 |
| 0.8 | 3 | 7.5 | 70.44 | 106.71 | 165.53 | 350.74 | 508.61 | 715.33 |
| 1 | 3 | 7.5 | 81.45 | 117.92 | 179.2 | 383.28 | 567.89 | 813.84 |
| 1.2 | 3 | 7.5 | 92.24 | 128.55 | 191.24 | 413.88 | 625.64 | 910.14 |
| 0.8 | 3.5 | 7.5 | 74.17 | 115.95 | 181.92 | 384.13 | 549.13 | 760.93 |
| 1 | 3.5 | 7.5 | 85.74 | 129.11 | 199.84 | 423.55 | 615.37 | 867.07 |
| 1.2 | 3.5 | 7.5 | 96.37 | 140.13 | 213.52 | 455.58 | 672.83 | 961.71 |
| 0.8 | 2.5 | 8 | 60.78 | 90.31 | 139.49 | 294.35 | 429.99 | 610.84 |
| 1 | 2.5 | 8 | 70.29 | 99.99 | 150.99 | 322.64 | 481.8 | 696.18 |
| 1.2 | 2.5 | 8 | 81.37 | 111.95 | 165.08 | 359.34 | 547.46 | 800.44 |
| 0.8 | 3 | 8 | 64.8 | 99.86 | 156.13 | 328.89 | 473.43 | 661.74 |
| 1 | 3 | 8 | 74.82 | 110.75 | 170.59 | 360.82 | 528.2 | 751.05 |
| 1.2 | 3 | 8 | 83.85 | 119.99 | 181.61 | 387.62 | 577.05 | 831.62 |
| 0.8 | 3.5 | 8 | 68.22 | 108.37 | 171.54 | 359.03 | 509.52 | 702.41 |
| 1 | 3.5 | 8 | 78.39 | 120.62 | 188.91 | 396.51 | 570.08 | 797.22 |
| 1.2 | 3.5 | 8 | 88.17 | 131.56 | 203.66 | 429.01 | 624.84 | 885.13 |
| 0.8 | 2.5 | 8.5 | 55.75 | 84.6 | 132.4 | 276.18 | 398.62 | 562.12 |
| 1 | 2.5 | 8.5 | 64.51 | 94.12 | 144.67 | 304.58 | 447.76 | 641.3 |
| 1.2 | 2.5 | 8.5 | 75.11 | 105.91 | 159.23 | 341.26 | 511.88 | 742.5 |
| 0.8 | 3 | 8.5 | 60.25 | 95.45 | 152.14 | 341.32 | 444.89 | 616.31 |
| 1 | 3 | 8.5 | 68.73 | 104.62 | 164.47 | 341.21 | 490.68 | 691.35 |
| 1.2 | 3 | 8.5 | 77.45 | 113.56 | 175.17 | 367.8 | 539.07 | 770.67 |
| 0.8 | 3.5 | 8.5 | 63.79 | 104.1 | 167.68 | 344.78 | 481.73 | 658.61 |
| 1 | 3.5 | 8.5 | 72.59 | 115.09 | 183.77 | 378.6 | 535.09 | 740.63 |
| 1.2 | 3.5 | 8.5 | 81.41 | 124.91 | 197 | 407.98 | 584.47 | 820.3 |

**Table A.1.** The natural frequency of PVS with checkerboard riffles arrangement (hole width b_h_=5mm; p_l_=30mm, n_rb_=16 pcs., n_rl_=21 pcs., l=0.2mm).

| **Surface thickness**  ***h_s_*, mm** | **Riffle radius *R_r_*, mm** | **Pitch of riffles arrangement *p_b_*, mm** | **Natural frequency, Hz** | | | | | |
| --- | --- | --- | --- | --- | --- | --- | --- | --- |
|  |  |  | **Mode № 1** | **Mode № 2** | **Mode № 3** | **Mode № 4** | **Mode № 5** | **Mode № 6** |
| 0.8 | 2.5 | 7.5 | 61.87 | 93.91 | 145.75 | 310.97 | 454 | 642.82 |
| 1 | 2.5 | 7.5 | 72.44 | 104.08 | 157.23 | 339.68 | 508.42 | 734.32 |
| 1.2 | 2.5 | 7.5 | 84.27 | 116.1 | 170.69 | 375.18 | 574.37 | 841.53 |
| 0.8 | 3 | 7.5 | 64.12 | 102.24 | 161.58 | 343.03 | 491.7 | 683.44 |
| 1 | 3 | 7.5 | 74.76 | 112.57 | 174.39 | 371.35 | 543.04 | 770.79 |
| 1.2 | 3 | 7.5 | 86.46 | 124.78 | 189.18 | 407.31 | 607.61 | 875.87 |
| 0.8 | 3.5 | 7.5 | 68.12 | 114.26 | 183.62 | 368.65 | 545.51 | 743.14 |
| 1 | 3.5 | 7.5 | 77.59 | 123.3 | 195.31 | 405.52 | 589.19 | 819.2 |
| 1.2 | 3.5 | 7.5 | 89.02 | 135.11 | 210.33 | 446.08 | 648.96 | 917.57 |
| 0.8 | 2.5 | 8 | 56.01 | 87.23 | 137.59 | 289.26 | 416.26 | 584.27 |
| 1 | 2.5 | 8 | 65.88 | 97.37 | 150.06 | 318.72 | 469.01 | 671 |
| 1.2 | 2.5 | 8 | 76.27 | 108.58 | 163.38 | 352.96 | 530.13 | 768.16 |
| 0.8 | 3 | 8 | 58.73 | 96.01 | 153.88 | 322.32 | 456.12 | 628.86 |
| 1 | 3 | 8 | 68.06 | 105.43 | 166.35 | 348.41 | 501.36 | 705.08 |
| 1.2 | 3 | 8 | 77.91 | 115.71 | 178.95 | 379.24 | 556.22 | 793.66 |
| 0.8 | 3.5 | 8 | 62.78 | 107.89 | 175.45 | 366 | 509.69 | 689.11 |
| 1 | 3.5 | 8 | 71.2 | 116.62 | 187.7 | 390.44 | 550.1 | 757 |
| 1.2 | 3.5 | 8 | 80.91 | 125.11 | 197.27 | 413.07 | 594.33 | 835.53 |
| 0.8 | 2.5 | 8.5 | 51.75 | 82.72 | 132.42 | 274.77 | 389.93 | 542.54 |
| 1 | 2.5 | 8.5 | 60.44 | 91.91 | 144.22 | 301.83 | 437.04 | 619.28 |
| 1.2 | 2.5 | 8.5 | 69.83 | 102.17 | 156.69 | 333.46 | 492.84 | 707.54 |
| 0.8 | 3 | 8.5 | 54.25 | 90.79 | 147.38 | 304.93 | 426.24 | 583.15 |
| 1 | 3 | 8.5 | 62.75 | 100.05 | 160.31 | 331.57 | 470.03 | 654.28 |
| 1.2 | 3 | 8.5 | 71.69 | 109.79 | 172.87 | 360.85 | 520.51 | 735.37 |
| 0.8 | 3.5 | 8.5 | 57.77 | 101.08 | 166.04 | 342.58 | 472.56 | 635.41 |
| 1 | 3.5 | 8.5 | 66.02 | 110.8 | 181.01 | 370.46 | 514.25 | 701.88 |
| 1.2 | 3.5 | 8.5 | 74.22 | 119.34 | 192.42 | 395.05 | 556.22 | 771.49 |

**Table A.2.** The natural oscillation frequency of PVS with row riffles arrangement (hole width b_h_=5mm; p_l_=30mm, n_rb_=16 pcs., n_rl_=21 pcs., l=0.2mm).

| **Surface thickness**  ***h_s_*, mm** | **Riffle radius *R_r_*, mm** | **Pitch of riffles arrangement *p_b_*, mm** | **Natural frequency, Hz** | | | | | |
| --- | --- | --- | --- | --- | --- | --- | --- | --- |
|  |  |  | **Mode № 1** | **Mode № 2** | **Mode № 3** | **Mode № 4** | **Mode № 5** | **Mode № 6** |
| 0.8 | 1.8 | 6 | 71.82 | 94.48 | 134.25 | 301.85 | 475.11 | 709.56 |
| 1 | 1.8 | 6 | 85.38 | 108.06 | 147.89 | 342.33 | 553.19 | 836.65 |
| 1.2 | 1.8 | 6 | 98.64 | 122.49 | 164.21 | 386.43 | 632.18 | 962.85 |
| 0.8 | 2.1 | 6 | 74.74 | 100.72 | 146.82 | 322.19 | 497.82 | 737.63 |
| 1 | 2.1 | 6 | 88.16 | 111.68 | 153.01 | 353.78 | 571.10 | 861.05 |
| 1.2 | 2.1 | 6 | 101.39 | 128.44 | 175.97 | 406.85 | 656.77 | 992.51 |
| 0.8 | 2.4 | 6 | 75.99 | 108.42 | 164.66 | 350.49 | 522.53 | 757.86 |
| 1 | 2.4 | 6 | 88.66 | 119.29 | 173.57 | 381.5 | 589.94 | 873.86 |
| 1.2 | 2.4 | 6 | 101.75 | 132.21 | 185.96 | 420.66 | 666.82 | 999.18 |
| 0.8 | 1.8 | 6.4 | 64.51 | 87.26 | 126.81 | 280.54 | 433.86 | 641.5 |
| 1 | 1.8 | 6.4 | 77.8 | 102.79 | 143.05 | 327.34 | 522.88 | 786.11 |
| 1.2 | 1.8 | 6.4 | 92.11 | 115.73 | 157.3 | 366.04 | 594.42 | 901.21 |
| 0.8 | 2.1 | 6.4 | 68.02 | 94.78 | 141.52 | 305.48 | 462.53 | 677.11 |
| 1 | 2.1 | 6.4 | 78.86 | 104.48 | 150.23 | 334.01 | 521.29 | 775.66 |
| 1.2 | 2.1 | 6.4 | 90.51 | 117.74 | 165.31 | 375.49 | 595.49 | 891.46 |
| 0.8 | 2.4 | 6.4 | 69.86 | 102.59 | 158.7 | 333.27 | 488.76 | 701.7 |
| 1 | 2.4 | 6.4 | 80.94 | 111.57 | 165.61 | 358.28 | 545.34 | 801.28 |
| 1.2 | 2.4 | 6.4 | 92.53 | 123.45 | 177.68 | 395.16 | 615.66 | 914.25 |
| 0.8 | 1.8 | 6.8 | 58.98 | 81.59 | 120.82 | 263.24 | 401.05 | 588.38 |
| 1 | 1.8 | 6.8 | 70.85 | 94.53 | 135.34 | 303.84 | 474.85 | 704.95 |
| 1.2 | 1.8 | 6.8 | 83.56 | 107.5 | 149.45 | 342.18 | 546.88 | 822.28 |
| 0.8 | 2.1 | 6.8 | 62.24 | 88.92 | 135.52 | 287.22 | 427.67 | 621.03 |
| 1 | 2.1 | 6.8 | 72.31 | 99.17 | 145.8 | 319.52 | 489.58 | 720.15 |
| 1.2 | 2.1 | 6.8 | 82.41 | 110.01 | 157.94 | 352.99 | 550.6 | 817.02 |
| 0.8 | 2.4 | 6.8 | 64.99 | 96.95 | 151.88 | 314.98 | 456.94 | 652.73 |
| 1 | 2.4 | 6.8 | 74.8 | 105.67 | 159.68 | 340.76 | 510.85 | 744.13 |
| 1.2 | 2.4 | 6.8 | 84.24 | 114.53 | 167.65 | 367.62 | 565.38 | 834.09 |

**Table A.3.** The natural oscillation frequency of PVS with checkerboard riffles arrangement (hole width b_h_=3.2mm; p_l_=30mm; n_rb_=19 pcs.; n_rl_=21 pcs.; l=0.1mm).

| **Surface thickness**  ***h_s_*, mm** | **Riffle radius *R_r_*, mm** | **Pitch of riffles arrangement *p_b_*, mm** | **Natural frequency, Hz** | | | | | |
| --- | --- | --- | --- | --- | --- | --- | --- | --- |
|  |  |  | **Mode № 1** | **Mode № 2** | **Mode № 3** | **Mode № 4** | **Mode № 5** | **Mode № 6** |
| 0.8 | 1.8 | 6 | 69.49 | 91.13 | 129.66 | 290.42 | 457.25 | 684.05 |
| 1 | 1.8 | 6 | 84.06 | 106.53 | 146.07 | 337.64 | 545.06 | 824.09 |
| 1.2 | 1.8 | 6 | 97.58 | 121.26 | 162.71 | 382.56 | 626.12 | 958.34 |
| 0.8 | 2.1 | 6 | 71.49 | 97.87 | 144.56 | 314.1 | 480.3 | 707.89 |
| 1 | 2.1 | 6 | 84.74 | 110.19 | 155.49 | 350.43 | 554.91 | 832.16 |
| 1.2 | 2.1 | 6 | 99.29 | 126.07 | 173.22 | 399.54 | 643.87 | 972.39 |
| 0.8 | 2.4 | 6 | 76.38 | 109.2 | 165.77 | 353.98 | 527.95 | 765.2 |
| 1 | 2.4 | 6 | 87.65 | 118.36 | 172.6 | 379.18 | 585.33 | 865.95 |
| 1.2 | 2.4 | 6 | 100.4 | 130.7 | 184.3 | 416.01 | 658.47 | 986.13 |
| 0.8 | 1.8 | 6.4 | 65.08 | 86.96 | 125.72 | 278.26 | 432.95 | 643.7 |
| 1 | 1.8 | 6.4 | 76.23 | 99.03 | 138.94 | 315.95 | 501.74 | 752.05 |
| 1.2 | 1.8 | 6.4 | 88.95 | 112.93 | 154.83 | 358.52 | 578.37 | 837.32 |
| 0.8 | 2.1 | 6.4 | 66.49 | 93.09 | 139.88 | 299.86 | 452.05 | 661.15 |
| 1 | 2.1 | 6.4 | 78.39 | 104.39 | 150.21 | 334.03 | 521.11 | 775.17 |
| 1.2 | 2.1 | 6.4 | 89.62 | 116.86 | 164.41 | 373.02 | 590.79 | 883.81 |
| 0.8 | 2.4 | 6.4 | 69.43 | 102.18 | 158.17 | 332.39 | 487.25 | 698.88 |
| 1 | 2.4 | 6.4 | 80.08 | 110.85 | 164.91 | 356.65 | 541.86 | 794.98 |
| 1.2 | 2.4 | 6.4 | 91.93 | 122.96 | 177.25 | 394.07 | 613.19 | 909.9 |
| 0.8 | 1.8 | 6.8 | 59.22 | 82.02 | 121.46 | 264.96 | 403.74 | 592.11 |
| 1 | 1.8 | 6.8 | 69.55 | 92.59 | 132.66 | 297.07 | 464.34 | 690.09 |
| 1.2 | 1.8 | 6.8 | 81.12 | 105.29 | 147.44 | 336.11 | 534.22 | 800.56 |
| 0.8 | 2.1 | 6.8 | 62.24 | 88.27 | 134.19 | 284.67 | 425.39 | 619.96 |
| 1 | 2.1 | 6.8 | 71.72 | 98.27 | 144.49 | 316.54 | 485.16 | 714.07 |
| 1.2 | 2.1 | 6.8 | 81.57 | 109.15 | 157.03 | 350.46 | 545.87 | 809.44 |
| 0.8 | 2.4 | 6.8 | 63.56 | 96.03 | 151.45 | 312.94 | 451.04 | 641.23 |
| 1 | 2.4 | 6.8 | 74.09 | 104.81 | 158.59 | 338.24 | 506.69 | 737.88 |
| 1.2 | 2.4 | 6.8 | 83.68 | 114.04 | 167.44 | 366.12 | 562.09 | 828.9 |

**Table A.4.** The natural oscillation frequency of PVS with row riffles arrangement (hole width b_h_=3.2mm, p_l_=30mm, n_rb_=19 pcs., n_rl_=21 pcs., l=0.1mm).

| **Surface thickness *h_s_*, mm** | **Pitch of riffles arrangement *p_b_*, mm** | **Natural frequency, Hz** | | | | | |
| --- | --- | --- | --- | --- | --- | --- | --- |
|  |  | **Mode № 1** | **Mode № 2** | **Mode № 3** | **Mode № 4** | **Mode № 5** | **Mode № 6** |
| hole width *b_h_*=5mm (*p_l_*=30mm, *n_rb_*=32 pcs., *n_rl_*=21 pcs., *l*=0mm) | | | | | | | |
| 0.8 | 7.5 | 53.87 | 66.88 | 89.52 | 210.03 | 342.2 | 516.76 |
| 1 | 7.5 | 67.04 | 83.08 | 111.17 | 260.49 | 424.71 | 641.75 |
| 1.2 | 7.5 | 80.13 | 99.12 | 132.57 | 310.24 | 506,17 | 765.38 |
| 0.8 | 8 | 47.18 | 61.01 | 84.74 | 194.09 | 308.19 | 458.68 |
| 1 | 8 | 58.71 | 75.82 | 105.32 | 240,87 | 382.55 | 569.66 |
| 1.2 | 8 | 70.16 | 90.48 | 125.69 | 287.07 | 456.04 | 679.48 |
| 0.8 | 8.5 | 41.94 | 56.46 | 81.03 | 181.52 | 281.3 | 412.92 |
| 1 | 8.5 | 52.19 | 70.19 | 100.78 | 225.4 | 349.27 | 512.91 |
| 1.2 | 8.5 | 62.37 | 83.79 | 120.35 | 268.79 | 416.48 | 611.87 |
| hole width *b_h_*=3.2mm (*p_l_*=30mm, *n_rb_*=38 pcs., *n_rl_*=21 pcs., *l*=0mm) | | | | | | | |
| 0.8 | 6 | 59.83 | 73.35 | 96.89 | 229.49 | 377.11 | 571.14 |
| 1 | 6 | 74.32 | 91.02 | 120.25 | 284.46 | 467.53 | 708.34 |
| 1.2 | 6 | 88.76 | 108.57 | 143.42 | 338.87 | 557.13 | 844.49 |
| 0.8 | 6.4 | 52.67 | 66.82 | 91.18 | 211.49 | 339.9 | 508.78 |
| 1 | 6.4 | 65.45 | 82.96 | 113.26 | 262.31 | 421.55 | 631.19 |
| 1.2 | 6.4 | 78.17 | 98.99 | 135.16 | 312.65 | 502.48 | 752.66 |
| 0.8 | 6.8 | 46.95 | 61.65 | 86.68 | 197.07 | 310.04 | 459.65 |
| 1 | 6.8 | 58.31 | 76.58 | 107.74 | 244.56 | 384.64 | 569.12 |
| 1.2 | 6.8 | 69.7 | 91.4 | 128.65 | 291.62 | 458.59 | 678.74 |

**Table A.5.** The natural oscillation frequency of PVS without riffles.
